# The fraction of varicella zoster virus-specific antibodies among all intrathecally-produced antibodies discriminates between patients with varicella zoster virus reactivation and multiple sclerosis

**DOI:** 10.1186/2045-8118-11-3

**Published:** 2014-02-12

**Authors:** Carolin Otto, Jörg Hofmann, Carsten Finke, Mathias Zimmermann, Klemens Ruprecht

**Affiliations:** 1 Department of Neurology, Charité – Universitätsmedizin Berlin, Charitéplatz 1, 10117 Berlin, Germany; 2 Labor Berlin, Charité-Vivantes GmbH and Institute for Virology, Charité – Universitätsmedizin Berlin, Charitéplatz 1, 10117 Berlin, Germany; 3 Labor Berlin, Charité-Vivantes GmbH and Institute of Laboratory Medicine, Clinical Chemistry, and Pathobiochemistry, Charité - Universitätsmedizin Berlin, Augustenburger Platz 1, 13353 Berlin, Germany; 4 Clinical and Experimental Multiple Sclerosis Research Center, Charité – Universitätsmedizin Berlin, Berlin, Germany

**Keywords:** Varicella zoster virus, Multiple sclerosis, Antibodies, Antibody index, Specific fraction, Cerebrospinal fluid

## Abstract

**Background:**

Primary infection with or reactivation of varicella zoster virus (VZV) can cause neurologic complications, which typically result in an intrathecal production of VZV-specific antibodies. Intrathecal antibodies to VZV are detectable by an elevated antibody index (AI). However, elevated VZV AIs are also found in more than half of patients with multiple sclerosis (MS), where they are thought to be part of a polyspecific intrathecal immune response. Determination of the fraction of intrathecally-produced virus-specific antibodies among all intrathecally produced antibodies may discriminate between virus-specific and polyspecific intrathecal immune responses, but the fraction of intrathecally-produced VZV-specific immunoglobulin (Ig)G of the total intrathecally produced IgG (*F*_S_ anti-VZV) in patients with MS and VZV reactivation has hitherto not been compared.

**Findings:**

*F*_S_ anti-VZV was calculated in patients with a clinically isolated syndrome suggestive of multiple sclerosis (MS) or MS (n = 20) and in patients with VZV reactivation (7 samples from 5 patients), which all had elevated VZV AIs. The median *F*_S_ anti-VZV was 35-fold higher in patients with VZV reactivation (45.1%, range 13.5-73%) than in patients with CIS/MS (1.3%, range 0.3-5.3%; *p* = 0.0001). While there was thus no overlap of *F*_S_ anti-VZV values between groups, VZV AIs completely overlapped in patients with CIS/MS (1.6-14.8) and VZV reactivation (2.1-8.1).

**Conclusions:**

The fraction of intrathecally-produced VZV-specific IgG of the total intrathecally produced IgG discriminates between patients with VZV reactivation and MS. Our results provide further evidence that intrathecally-produced VZV antibodies are part of the polyspecific immune response in patients with MS.

## Introduction

Both primary infection and reactivation of varicella zoster virus (VZV) can cause various neurologic complications, including meningitis, meningoencephalitis, cerebellitis, myelitis, and cerebral vasculitis [1,2]. These conditions typically lead to an intrathecal production of VZV-specific antibodies [3,4]. Intrathecally-produced VZV antibodies can be detected by an elevated antibody index (AI), which is the ratio between the CSF/serum quotient of virus-specific antibodies (Q_spec_) and the CSF/serum quotient for total IgG (Q_IgG_) [4]. However, elevated VZV AIs are also present in more than half of patients with multiple sclerosis (MS), a chronic inflammatory demyelinating central nervous system (CNS) disease, where they are thought to be part of a polyspecific intrathecal immune response [5]. To characterize intrathecal antiviral immune responses in detail, but also for clinical differential diagnostic purposes, it appears desirable to clarify whether elevated antiviral AIs reflect virus-driven or polyspecific intrathecal immune responses. Others and we have previously shown that this can be achieved by calculating the fraction (*F*_S_) of the intrathecally-synthesized virus-specific immunoglobulin (Ig)G of the total intrathecally-synthesized IgG [5-7]. Nevertheless, the fraction of intrathecally-produced VZV-specific IgG of the total intrathecally-produced IgG (*F*_S_ anti-VZV) in patients with VZV reactivation and MS has hitherto not been compared. We herein show that calculation of *F*_S_ anti-VZV allows discrimination between patients with VZV reactivation and MS.

## Patients and methods

### Patients

The study was approved by the institutional review board Charité – Universitätsmedizin Berlin (EA1/068/12). All lumbar punctures were performed for diagnostic purposes only, with the patients’ written informed consent. Twenty patients (14 female, 6 male; median [range] age 33 [21-60] years) with an elevated VZV AI (defined as VZV AI ≥ 1.5) and either a clinically isolated syndrome (CIS, n = 11) suggestive of MS, relapsing-remitting MS (n = 8), or primary progressive MS (n = 1) according to the McDonald 2005 criteria [8] were included in the study. All CIS/MS patients had an intrathecal IgG synthesis, demonstrated by the presence of CSF-specific oligoclonal bands. We defined CSF/serum samples obtained up to 4 weeks after the onset of a clinical relapse as samples taken during a relapse (n = 14) and CSF/serum samples obtained more than 4 weeks after a clinical relapse as samples taken during remission (n = 6).

We identified 13 patients with CNS complications of VZV reactivation, confirmed by PCR detection of VZV DNA in CSF. Seven CSF/serum samples from five of these patients (2 female, 3 male) met the requirements for determination of *F*_S_, i.e. an intrathecal IgG synthesis as proven by Q_IgG_ > Q_mean_ and an intrathecal VZV-specific antibody synthesis (VZV AI ≥ 1.5). The median (range) age of these five patients was 68 (45-74) years and their CSF/serum samples were drawn 19 (median, range; 2-89) days after clinical onset of symptoms; clinical diagnoses were VZV meningitis, VZV meningitis/Ramsay Hunt syndrome, VZV meningitis/retinitis, VZV encephalitis/vitritis, and VZV-associated cerebral vasculopathy. We additionally analysed two patients (1 female, 1 male; 67 and 54 years) with herpes simplex virus (HSV) encephalitis, proven by PCR detection of HSV DNA in CSF, who had a Q_IgG_ > Q_mean_, and an intrathecal VZV-specific antibody synthesis (VZV AIs 2 and 10.1).

Serum and cerebrospinal fluid (CSF) samples collected between April 2004 and July 2012 and stored at -20°C, were available from all patients. To exclude a confounding influence of artificial blood contamination on antibody measurements, only CSF samples with an erythrocyte count of < 400/μl were included [9].

### Measurement and calculation of virus-specific antibody indices

Total albumin and IgG concentrations in serum and CSF were measured nephelometrically (BN ProSpec, Siemens Healthcare Diagnostics, Eschborn, Germany). AIs for antibodies against VZV were determined exactly as previously described by enzyme-linked immunosorbent assays (Enzygnost, Siemens Healthcare Diagnostics) [7]. A standard curve consisting of serial dilutions of standard human plasma (SHP, Siemens Healthcare Diagnostics) was included on each ELISA plate. The same batch of SHP was used for all experiments. AI values were calculated either by the formula: AI = (IgG_spec_ CSF/IgG_spec_ serum)/(IgG_total_ CSF/IgG_total_ serum) = (quotient CSF/serum specific IgG)/(quotient CSF/serum total IgG) = Q_spec_/Q_IgG_ if Q_IgG_ < Q_Lim,_ or AI = Q_spec_/Q_Lim_ if Q_IgG_ > Q_Lim_[4]. Q_Lim_ was calculated as described [3].

### Calculation of the intrathecal fraction of VZV-specific antibodies

The fraction (in %) of the intrathecally-synthesized VZV-specific IgG concentration of the total intrathecally-synthesized IgG concentration (*F*_S_ anti-VZV) was calculated as described [6,7]. Calculation of *F*_S_ anti-VZV requires determination of the absolute concentration (in μg/ml) of VZV-specific IgG antibodies in serum and CSF. Therefore, samples must be tested in parallel with a standard curve, for which we used SHP, with a known concentration of VZV-specific IgG. Applying a previously-described method [7], we determined the absolute concentration of anti-VZV antibodies in the SHP used in this study to be 24 μg/ml.

*F*_S_ values were determined as ratio of the intrathecally-produced concentration of virus-specific antibodies (AB_Loc_) and the concentration of intrathecally-synthesized total IgG (IgG_Loc_). As previously described [6], for comparison of means in different groups *F*_S_ refers to Q_mean_ instead of Q_Lim_. Therefore, *F*_S_ was calculated by the formula: *F*_S_ = AB_Loc_ (mean)/IgG_Loc_ (mean) × 100 [%], with IgG_Loc_ (mean) = (Q_IgG_ – Q_mean_) × IgG(serum) [mg/l], AB_Loc_ (mean) = (Q_spec_ - Q_mean_) × AB(serum) [mg/l], and Q_mean_ (IgG) = (0.65 (Q_Alb_^2^ + 8)^0.5^ – 1.4) × 10^-3^[6]. Of note, *F*_S_ can only be calculated for Q_IgG_ > Q_mean_ and in cases of a proven intrathecal virus-specific antibody synthesis, as defined by an AI ≥ 1.5 [4].

### Statistics

Statistical significance of different *F*_S_ values in patients with CIS/MS and VZV reactivation and in CIS/MS patients in relapse and remission was assessed by Mann–Whitney *U* test using GraphPad Prism 5.04 software.

## Findings

**Figure 1:**
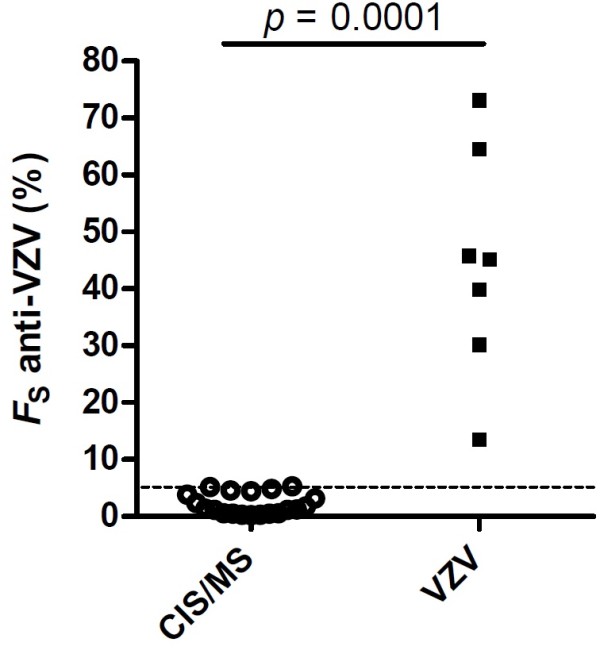
**Percentage of VZV-specific intrathecally-produced IgG of the total intrathecally-produced IgG (*F***_**S **_**anti-VZV) in patients with a clinically isolated syndrome or multiple sclerosis (CIS/MS) and VZV reactivation with central nervous system complications (VZV).** An *F*_S_ anti-VZV of 5% is indicated by the dashed line. Statistical significance of the difference between groups was assessed by Mann–Whitney *U* test.

The median *F*_S_ anti-VZV in the 20 patients with CIS/MS was 1.3%, indicating that in these patients 1.3% (median) of all intrathecally produced antibodies were directed against VZV (Table 1, Figure 1). In contrast, median *F*_S_ anti-VZV in the 7 CSF/serum pairs from 5 patients with VZV reactivation was 45.1% (VZV meningitis, 13.5%; VZV meningitis/Ramsay Hunt syndrome, 30.2% and 39.8%; VZV meningitis/retinitis, 45.1%; VZV encephalitis/vitritis, 73% and 45.8%; VZV-associated cerebral vasculopathy, 64.6%). Thus, the median amount of intrathecally-synthesized anti-VZV antibodies was 35-fold higher in patients with VZV reactivation compared to patients with CIS/MS (*p* = 0.0001). There was no overlap of *F*_S_ anti-VZV values between patients with CIS/MS and VZV reactivation. Conversely, VZV AI values overlapped between both groups, re-confirming that AIs do not discriminate between a virus-driven and a polyspecific intrathecal immune response. A comparison of *F*_S_ anti-VZV between CIS/MS patients in relapse and remission revealed no significant difference (*p* = 0.34). To confirm the specificity of elevated *F*_S_ anti-VZV values for VZV reactivation we additionally determined *F*_S_ anti-VZV in 2 patients with HSV encephalitis with a concomitant intrathecal VZV antibody synthesis. *F*_S_ anti-VZV values of these 2 patients (0.3% and 4.96%) were in the same range as those of patients with CIS/MS.

**Table 1:** Intrathecal VZV-specific immune responses in patients with CIS/MS and VZV reactivation

	**IgG**_**Loc **_**(mean) (mg/l)**	**AB**_**Loc **_**(mean) (mg/l)**	**AB (serum) (mg/l)**	**AI**	***F***_**S **_anti-VZV (%)**
CIS/MS (n = 20)	44.2 (5.6-103.1)	0.5 (0.1-4.5)	51 (7.6-353)	3.9 (1.6-14.8)	1.3 (0.3-5.3)
VZV (n = 7)*	20.7 (5.2-89.2)	8 (0.7-40.2)	244 (42.4-962)	6.9 (2.1-8.1)	45.1 (13.5-73)

Median (range) of intrathecally-synthesized total IgG (IgG_Loc_ (mean)), intrathecally-synthesized VZV-specific IgG (AB_Loc_ (mean)), anti-VZV antibody concentration in serum (AB (serum)), antibody index (AI), and intrathecally-produced fraction of VZV-specific antibodies (*F*_S_) in patients with a clinically isolated syndrome or multiple sclerosis (CIS/MS) and VZV reactivation with central nervous system complications (VZV).

*7 CSF/serum samples from 5 patients.

**p = 0.0001 (CIS/MS vs. VZV).

## Discussion

This study shows that *F*_S_ anti-VZV is about 35-fold higher in patients with VZV reactivation than in patients with CIS/MS. *F*_S_ anti-VZV values did not overlap between both groups, suggesting that determination of *F*_S_ anti-VZV can accurately discriminate between a polyspecific immune response in CIS/MS and a virus-specific immune response in VZV reactivation. This is in accordance with previous reports demonstrating that patients with HSV encephalitis, subacute sclerosing panencephalitis (SSPE), or Epstein-Barr virus (EBV) replication in the CNS have a fraction of the intrathecally-produced virus-specific IgG that is 20- to 60-fold higher than in patients with MS [6,7]. In HSV encephalitis, SSPE, or EBV replication in the CNS a median of 9%, 19%, and 28% of the total intrathecally synthesized IgG was found to be directed against HSV, measles virus, or EBV, respectively [6,7]. The median *F*_S_ anti-VZV observed in this work was even higher (45.1%), indicating that VZV reactivation with CNS complications results in a highly-focussed VZV-specific intrathecal antibody response. Consistent with previous observations in 4 patients with HSV encephalitis [6], the *F*_S_ anti-VZV values determined in 2 patients with HSV encephalitis in this work were in the same range as those of CIS/MS patients, suggesting that intrathecal production of VZV antibodies represents a formerly-recognized non-specific anti-VZV antibody synthesis in patients with HSV encephalitis [3] and that the levels of *F*_S_ anti-VZV elevation in patients with VZV reactivation observed in the present study might be specific for this condition. Nevertheless, further studies involving larger patient numbers will be required to establish more definitively the specificity of elevated *F*_S_ anti-VZV values for VZV reactivation and to determine a cut-off above which elevated *F*_S_ anti-VZV values can be considered specific for VZV reactivation.

The *F*_S_ anti-VZV values in patients with CIS/MS were in the same range as previously reported *F*_S_ values for VZV, HSV, measles virus, rubella virus, and EBV in patients with CIS/MS [6,7]. The similar *F*_S_ values for VZV, HSV, measles virus, rubella virus, and EBV in patients with CIS/MS as well as the marked difference in *F*_S_ anti-VZV between patients with CIS/MS and VZV reactivation, strongly suggest that the intrathecal production of VZV antibodies is part of the polyspecific intrathecal humoral immune response in MS. Our finding that *F*_S_ anti-VZV values did not differ between CIS/MS patients in relapse and remission does not support the idea that *F*_S_ anti-VZV could be influenced by clinical disease activity.

A previous study reported the presence of VZV DNA in CSF of patients with MS during relapses, leading to the proposal of a participation of VZV in the pathogenesis of MS [10,11]. However, those findings could not be reproduced [12]. Likewise, our results indicating that VZV antibodies are part of the polyspecific intrathecal immune response in MS argue against the presence of VZV in the CSF of the CIS/MS patients investigated in this study.

An intrathecal IgG synthesis is found in ~95% of patients with MS [5]. Together with previous work [6,7], our results underscore that this intrathecally produced IgG is indeed polyspecific with only a small proportion of the intrathecally produced IgG being directed against each particular antigen target. The fact that this is already evident in patients with a CIS is compatible with the concept that a variety of different plasma cell clones populate the CNS early during the development of MS.

One limitation of this study is its relatively small patient numbers. Especially, the number of patients with VZV reactivation was limited as less than half of these patients met the requirements for determination of *F*_S_ anti-VZV (Q_IgG_ > Q_mean_ and VZV AI ≥1.5). Nevertheless, the difference between both groups was clear-cut, highly significant, and corresponded well to previous findings on the intrathecal production of antiviral antibodies in MS.

## Conclusions

Altogether, our study demonstrates that determination of *F*_S_ anti-VZV allows discrimination between patients with VZV reactivation and CIS/MS. It also provides further evidence that intrathecally produced VZV antibodies are part of the polyspecific immune response in MS. Determination of *F*_S_ can be applied to any intrathecally produced microorganism- or antigen-specific antibody and may therefore prove a useful tool to differentiate between microorganism/antigen-triggered and polyspecific intrathecal humoral immune responses in different contexts and diseases.

## Competing interests

CO, JH, CF, and MZ report no disclosures. KR has received speaker’s honoraria and travel grants from Merck Serono, Teva, Biogen Idec, Novartis Pharma GmbH, and Bayer Schering as well as scientific grant support from Novartis Pharma GmbH. KR is supported by the German ministry of education and research (BMBF/KKNMS, Competence Network Multiple Sclerosis).

## Authors’ contributions

CO participated in the design of the study, carried out the experiments, and collected and analyzed data, JH participated in the design of the study and analyzed data, CF and MZ collected and analyzed data, KR conceived of the study, participated in the design of the study, analyzed data, and wrote the manuscript. All authors read and approved the manuscript.
